# Supplementary material for: Influenza A Virus Infection Reactivates Human Endogenous Retroviruses Associated with Modulation of Antiviral Immunity
Source: Viruses. 2022 Jul 21;14(7):1591. doi: 10.3390/v14071591 (PMC9320126; doi:10.3390/v14071591)
Supplement: Supplementary file 1 [file viruses-14-01591-s001.zip › MotifAnalysis.zip!/MotifOutput_PR8M/homerResults.html]

MotifOutput// - Homer de novo Motif Results


# Homer *de novo* Motif Results (MotifOutput//)

Known Motif Enrichment Results  
Gene Ontology Enrichment Results  
If Homer is having trouble matching a motif to a known motif, try copy/pasting the matrix file into
STAMP  
More information on motif finding results: HOMER
| Description of Results
| Tips
  
Total target sequences = 23  
Total background sequences = 45868  
\* - possible false positive  

|  |  |  |  |  |  |  |  |  |
| --- | --- | --- | --- | --- | --- | --- | --- | --- |
| Rank | Motif | P-value | log P-pvalue | % of Targets | % of Background | STD(Bg STD) | Best Match/Details | Motif File |
| 1 | A C G T A G T C A C G T A C G T A C T G A C G T A C T G A C T G C T G A C T G A | 1e-21 | -4.956e+01 | 56.52% | 0.76% | 45.9bp (78.9bp) | CEBPD/MA0836.2/Jaspar(0.704) More Information \| Similar Motifs Found | motif file (matrix) |
| 2 | G T C A C G T A A T C G C G T A A G T C C G A T G T A C G A C T C T A G G T A C C G A T G T A C | 1e-21 | -4.871e+01 | 26.09% | 0.01% | 13.5bp (42.3bp) | PB0099.1\_Zfp691\_1/Jaspar(0.598) More Information \| Similar Motifs Found | motif file (matrix) |
| 3 | C T A G A C T G A C G T A G T C C G T A A G T C C T A G A G T C A C G T A G T C A G T C A C G T | 1e-21 | -4.871e+01 | 26.09% | 0.00% | 14.7bp (51.8bp) | PAX5/MA0014.3/Jaspar(0.823) More Information \| Similar Motifs Found | motif file (matrix) |
| 4 | A C T G A C G T A G T C C G T A A C T G A C T G A G T C A G T C A G T C A C G T A G T C A G T C | 1e-18 | -4.324e+01 | 21.74% | 0.00% | 11.5bp (49.1bp) | PAX5/MA0014.3/Jaspar(0.633) More Information \| Similar Motifs Found | motif file (matrix) |
| 5 | C G T A A C G T A G T C A G T C C T A G A C T G C G T A A C T G A C T G A G T C A G T C A C G T | 1e-18 | -4.212e+01 | 26.09% | 0.01% | 13.2bp (60.4bp) | ZFX(Zf)/mES-Zfx-ChIP-Seq(GSE11431)/Homer(0.727) More Information \| Similar Motifs Found | motif file (matrix) |
| 6 | A C T G C G T A A C G T A C T G A G T C A C G T A C T G A C G T A C T G A G T C A C G T A C G T | 1e-17 | -3.977e+01 | 21.74% | 0.01% | 5.5bp (20.2bp) | Gfi1b/MA0483.1/Jaspar(0.698) More Information \| Similar Motifs Found | motif file (matrix) |
| 7 | C G A T A G T C A G T C C G T A A G T C A G T C A C G T A G T C A C G T A C G T A C T G A C G T | 1e-16 | -3.849e+01 | 26.09% | 0.03% | 41.6bp (79.1bp) | SCRT1/MA0743.2/Jaspar(0.687) More Information \| Similar Motifs Found | motif file (matrix) |
| 8 | A C T G A C G T A G T C A G T C C T A G A G T C A G C T A C G T C G A T A G T C C G T A A C G T | 1e-15 | -3.519e+01 | 21.74% | 0.01% | 15.9bp (39.3bp) | HINFP(Zf)/K562-HINFP.eGFP-ChIP-Seq(Encode)/Homer(0.672) More Information \| Similar Motifs Found | motif file (matrix) |
| 9 | C T A G A C G T C T G A C G T A A C T G A C G T A C G T A C G T C G T A C T A G C G T A A C T G | 1e-14 | -3.381e+01 | 26.09% | 0.05% | 33.1bp (55.9bp) | VDR/MA0693.2/Jaspar(0.571) More Information \| Similar Motifs Found | motif file (matrix) |
| 10 | C G T A A C G T C G T A A G T C C G T A A G T C A G T C C G T A C G T A A C G T A G T C A C T G | 1e-14 | -3.242e+01 | 30.43% | 0.17% | 47.1bp (93.2bp) | NFYA/MA0060.3/Jaspar(0.628) More Information \| Similar Motifs Found | motif file (matrix) |
| 11 | C T A G A C G T C T G A A C G T A G T C A C G T C G T A A C T G A G T C A C G T T C G A G T A C | 1e-14 | -3.230e+01 | 34.78% | 0.35% | 55.8bp (95.5bp) | Smad4/MA1153.1/Jaspar(0.649) More Information \| Similar Motifs Found | motif file (matrix) |
| 12 | A C G T A C G T A C T G A C G T C T G A C G T A C T G A A G T C C T G A A G T C C G T A A G T C | 1e-13 | -3.162e+01 | 34.78% | 0.38% | 46.1bp (99.6bp) | FOXP1/MA0481.3/Jaspar(0.702) More Information \| Similar Motifs Found | motif file (matrix) |
| 13 | A C G T A C G T A C T G A C T G C G T A A C T G C G T A C T G A A G T C A G T C A C G T A C G T | 1e-12 | -2.945e+01 | 34.78% | 0.50% | 56.0bp (87.4bp) | HSF1/MA0486.2/Jaspar(0.648) More Information \| Similar Motifs Found | motif file (matrix) |
| 14 | A G T C T G A C C T A G A C T G A C T G C G T A C G T A C G T A A C T G A C T G A C T G C G T A | 1e-12 | -2.908e+01 | 21.74% | 0.04% | 9.9bp (70.2bp) | Stat4/MA0518.1/Jaspar(0.641) More Information \| Similar Motifs Found | motif file (matrix) |
| 15 | A G T C A C G T A C T G A C T G A C T G A G T C A C G T A G T C A G T C A C G T A C T G C G T A | 1e-12 | -2.826e+01 | 21.74% | 0.05% | 14.5bp (118.4bp) | ZNF416(Zf)/HEK293-ZNF416.GFP-ChIP-Seq(GSE58341)/Homer(0.644) More Information \| Similar Motifs Found | motif file (matrix) |
| 16 | A G T C A C G T A C G T C G T A A C T G A G T C C T G A A C T G C G T A A G T C | 1e-12 | -2.802e+01 | 21.74% | 0.05% | 6.0bp (114.6bp) | NFIC/MA0161.2/Jaspar(0.651) More Information \| Similar Motifs Found | motif file (matrix) |
| 17 | A G T C C T G A C T A G A C T G A C T G G T C A A C G T A C G T C T A G A C G T | 1e-12 | -2.769e+01 | 39.13% | 1.03% | 46.0bp (92.3bp) | MF0011.1\_HMG\_class/Jaspar(0.672) More Information \| Similar Motifs Found | motif file (matrix) |
| 18 \* | A G T C C G T A C T G A A C G T A G T C C T A G C T A G A G T C C G T A A G T C A G C T A G T C | 1e-11 | -2.741e+01 | 30.43% | 0.34% | 43.3bp (88.7bp) | NFIA/MA0670.1/Jaspar(0.622) More Information \| Similar Motifs Found | motif file (matrix) |
| 19 \* | A C T G A C G T A C G T A C G T A C T G A C G T A C T G C G T A | 1e-11 | -2.703e+01 | 34.78% | 0.67% | 12.6bp (72.5bp) | POL007.1\_BREd/Jaspar(0.646) More Information \| Similar Motifs Found | motif file (matrix) |
| 20 \* | A G T C C G T A A C G T A C T G A C G T A C G T A G T C A G T C C G T A A C G T | 1e-11 | -2.677e+01 | 21.74% | 0.06% | 10.9bp (59.7bp) | NFATC4/MA1525.1/Jaspar(0.761) More Information \| Similar Motifs Found | motif file (matrix) |
| 21 \* | A C T G A G T C A G T C A G T C A C T G A G T C C G T A A C T G A C G T A C G T | 1e-9 | -2.264e+01 | 13.04% | 0.01% | 7.4bp (41.4bp) | THAP1/MA0597.1/Jaspar(0.669) More Information \| Similar Motifs Found | motif file (matrix) |
| 22 \* | G A C T A C G T A G T C A G T C A G C T A C T G C A G T C G T A A G T C A G C T G T C A G T A C | 1e-9 | -2.258e+01 | 26.09% | 0.34% | 32.2bp (77.4bp) | IKZF1/MA1508.1/Jaspar(0.711) More Information \| Similar Motifs Found | motif file (matrix) |
| 23 \* | A G C T A C G T A G C T T C G A A T G C A G C T A C G T C T A G C G A T G T A C | 1e-9 | -2.252e+01 | 34.78% | 1.19% | 39.6bp (71.3bp) | ZBTB7B/MA0694.1/Jaspar(0.624) More Information \| Similar Motifs Found | motif file (matrix) |
| 24 \* | C G T A A C T G C G T A C G T A A C G T A G T C A C G T A C T G A G T C A G T C A C G T A G T C | 1e-9 | -2.130e+01 | 17.39% | 0.05% | 25.5bp (88.7bp) | ASCL1(var.2)/MA1631.1/Jaspar(0.565) More Information \| Similar Motifs Found | motif file (matrix) |
| 25 \* | G T C A A C G T A G T C A C G T A T G C C G T A A C G T A C G T A C T G A G T C | 1e-8 | -1.951e+01 | 21.74% | 0.26% | 22.5bp (67.8bp) | Hoxa10(Homeobox)/ChickenMSG-Hoxa10.Flag-ChIP-Seq(GSE86088)/Homer(0.692) More Information \| Similar Motifs Found | motif file (matrix) |
| 26 \* | A G T C C G T A A G T C A G T C A C G T A C T G A G T C A G T C A G T C C G T A A C T G A C G T | 1e-8 | -1.934e+01 | 13.04% | 0.01% | 13.1bp (36.3bp) | ASCL1(var.2)/MA1631.1/Jaspar(0.778) More Information \| Similar Motifs Found | motif file (matrix) |
| 27 \* | A C T G A G T C A G T C A G T C A C G T A C G T A G T C A C G T A C G T A G T C A G T C A G T C | 1e-8 | -1.934e+01 | 13.04% | 0.02% | 13.1bp (39.5bp) | PB0124.1\_Gabpa\_2/Jaspar(0.709) More Information \| Similar Motifs Found | motif file (matrix) |
| 28 \* | C G T A C G T A G C T A A C G T C G T A A C G T A G T C A C G T A T G C A T G C | 1e-7 | -1.664e+01 | 21.74% | 0.45% | 26.8bp (70.2bp) | PB0126.1\_Gata5\_2/Jaspar(0.763) More Information \| Similar Motifs Found | motif file (matrix) |
| 29 \* | C G T A C T G A A G T C A C G T A G T C C G A T A C T G C T A G A C G T A C T G | 1e-7 | -1.624e+01 | 17.39% | 0.18% | 13.1bp (65.2bp) | ZEB1/MA0103.3/Jaspar(0.672) More Information \| Similar Motifs Found | motif file (matrix) |
| 30 \* | A C T G A G T C C G T A A G T C A G T C A G T C A C G T A C T G | 1e-7 | -1.623e+01 | 26.09% | 1.00% | 51.8bp (73.0bp) | CTCFL/MA1102.2/Jaspar(0.758) More Information \| Similar Motifs Found | motif file (matrix) |
| 31 \* | A C T G C G T A C G T A A C T G A T C G A G T C C G T A A C G T A G T C A C G T | 1e-5 | -1.340e+01 | 17.39% | 0.37% | 39.7bp (65.0bp) | ZNF528(Zf)/HEK293-ZNF528.GFP-ChIP-Seq(GSE58341)/Homer(0.685) More Information \| Similar Motifs Found | motif file (matrix) |
| 32 \* | C A G T A C G T C T G A G A C T G C A T C G T A C A T G A C T G A G T C A T G C G A C T C G T A | 1e-5 | -1.225e+01 | 26.09% | 2.00% | 44.1bp (66.3bp) | ZNF711(Zf)/SHSY5Y-ZNF711-ChIP-Seq(GSE20673)/Homer(0.657) More Information \| Similar Motifs Found | motif file (matrix) |
